# Simulated resections and RNS placement can optimize post-operative seizure outcomes when guided by fast ripple networks

**DOI:** 10.1101/2024.03.26.24304802

**Published:** 2024-03-28

**Authors:** Shennan Aibel Weiss, Michael R. Sperling, Jerome Engel, Anli Liu, Itzhak Fried, Chengyuan Wu, Werner Doyle, Charles Mikell, Sima Mofakham, Noriko Salamon, Myung Shin Sim, Anatol Bragin, Richard Staba

**Affiliations:** 1 Dept. of Neurology, State University of New York Downstate, Brooklyn, New York 11203, USA; 2 Dept. of Physiology and Pharmacology, State University of New York Downstate, Brooklyn, New York 11203, USA; 3 Dept. of Neurology, New York City Health + Hospitals/Kings County, Brooklyn, NY, 11203 USA; 4 Dept. of Neurology, Thomas Jefferson University, Philadelphia, PA 19107, USA; 5 Dept. of Neuroradiology, Thomas Jefferson University, Philadelphia, PA, 19107, USA; 6 Dept. of Neurosurgery, Thomas Jefferson University, Philadelphia, PA 19107, USA; 7 Dept. of Neurology, David Geffen School of Medicine at UCLA, Los Angeles, California 90095, USA; 8 Dept. of Neurosurgery, David Geffen School of Medicine at UCLA, Los Angeles, California 90095, USA; 9 Dept. of Neurobiology, David Geffen School of Medicine at UCLA, Los Angeles, California 90095, USA; 10 Dept. of Psychiatry and Biobehavioral Sciences, David Geffen School of Medicine at UCLA, Los Angeles, California 90095, USA; 11 Brain Research Institute, David Geffen School of Medicine at UCLA, Los Angeles, California 90095, USA; 12 Dept. of Neuroradiology, David Geffen School of Medicine at UCLA, Los Angeles, California 90095, USA; 13 Dept. of Medicine, David Geffen School of Medicine at UCLA, Los Angeles, California 90095, USA; 14 Department of Neurology, NYU Grossman School of Medicine, New York, NY, 10016 USA; 15 Department of Neurosurgery, NYU Grossman School of Medicine, New York, NY, 10016 USA; 16 Neuroscience Institute, NYU Langone Medical Center, New York, NY, 10016 USA; 17 Department of Neurosurgery, State University of New York Stony Brook, Stony Brook, New York 11790, USA

**Keywords:** Epilepsy surgery, neuromodulation, high-frequency oscillation, fast ripple, seizure onset zone, virtual resection

## Abstract

Resecting cortical tissue generating high-frequency oscillations (HFOs) has been investigated as a more efficacious alternative to resecting the clinically defined seizure onset zone (SOZ). In this study, we asked if seizure freedom would be achieved using virtual resections of fast ripple (FR) networks. We compared these virtual resections to the individual patient’s actual resection and clinical outcome. We conclude that the SOZ is the minimum territory of cortex that must be resected to achieve seizure freedom. By utilizing support vector machines (SVMs) with an accuracy of 0.78 for labeling seizure freedom using factors from FR networks we could predict whether resection of the SOZ corresponded with a seizure free outcome. Furthermore, this approach could identify regions that generate FR autonomously and at high rates outside the SOZ. In the patients who experienced seizures after resection of the SOZ, virtual resections that included the SOZ and other FR generating regions rendered the patient virtually seizure free. We examined responsive neurostimulator system (RNS) patients and virtually targeted the RNS stimulation contacts proximal to sites generating FR. We used the simulations to investigate if the likelihood of a RNS super responder (>90% seizure reduction) outcome would be increased.

## Introduction

The gold standard of epilepsy surgery is resection of the seizure onset zone (SOZ)^1^. Even if the seizure onset zone (SOZ) is sufficiently sampled by the stereo EEG (SEEG) implant, resection of the SOZ does not correlate well with seizure outcome^2–7^, and thus a considerable percentage of patients that undergo epilepsy surgery are not rendered seizure free^8–10^. Fast ripples (FR) are brief (8–50 ms) bursts of oscillatory (200–600 Hz) activity that are, in most context, pathological^11–14^. FRs are best recorded with stereo EEG electrodes during non-REM sleep. Analyzing hours of sleep improves the accuracy of localizing epileptogenic regions^15^. In retrospective studies, resecting 60% of FR events (i.e., FR resection ratio [RR]) had a 70–80% accuracy for predicting seizure freedom^15^.

We developed a novel measure of the residual FR generating activity territory following a resection. This measure is calculated using graph theoretical analysis^16^ and is named the rate-distance radius resected difference (RDRRD)^17^. For simplicity, we refer to the RDRRD as spatial FRnet. Spatial FRnet classified post-operative seizure-free patients more accurately than the FR RR^18^.

Past investigations have also asked if the sites that generate FR events at the highest rates must be resected to achieve seizure freedom^19,20^. This premise suggests certain sites in the FR network may be more important than others. To better investigate this possibility, we utilized FR onset times to calculate FR mutual information (MI) between FR nodes (*i.e.,* SEEG contacts). The FR MI value weighted the edges of the FR network. We then used this FR MI temporal correlational network to derive global and local graph measures. We found that a FR MI network with a longer path length correlated with a failure to improve after surgery^17^, and a failure to resect nodes generating FR at high rates and desynchronously (*i.e.,* autonomously) with other nodes correlated with a failure to achieve seizure freedom^18^. To account for these observations, we calculated two FR temporal graph theoretical metrics: 1) the path length resection ratio ( RR); and 2) unresected mean local efficiency (urmLE)^18^. Herein, these metrics are referred to as temporal FRnet-A and B^18^

Utilizing spatial and temporal FRnets to plan a resection raises many important questions: 1) Are hypothetical resections that target FR nets smaller or larger than resections that target the SOZ? 2) would such resections also encompass the SOZ? 3) Do effective epilepsy surgeries also require resecting other types of high-frequency oscillation (HFO) generating sites, such as ripples on spikes (RonS)? 4) Should a future clinical trial compare resection of the SOZ to FR nets? or should FR nets be used to tailor resection of the SOZ to include larger portions of the FR net.

This computational modeling study trained support vector machines (SVMs) using the FR measures, derived from the actual resections, as factors to label patients as seizure free. We then tested the SVM to label the seizure free outcome using FR measure factors derived from iterative virtual resections that targeted key nodes of the FR network. We compared the actual resection volumes to the virtual resection volumes.

Additionally, we examined patients implanted with the RNS device, and asked if alternate placement of the responsive neurostimulator (RNS) stimulation contacts at sites generating FR at high rates may correspond with possible improved seizure outcome. To approximate the brain regions that were maximally stimulated by the actual and virtual RNS placements, we defined the pre-implant SEEG electrode stimulated contacts as within a radius of <1.5 cm of the eight RNS contacts (i.e., two leads of either a four-contact depth or subdural strip)^21^. Our justification for using this method was that RNS stimulation intensities typically ranged from ~1–3 mAmps and monopolar stimulation would produce an electric field of ~2–4 mV/mm at 1.5 cm away from the stimulation source^22^. Since electric field strengths of at least 2–4 mV/mm can induce spike field coherence^23,24^, and smaller fields at distances greater than 1.5 cm may only influence spike timing^25^. We next calculated the SOZ stimulation ratio (SR), FR SR, and the FR stimulated global efficiency (SGe), herein described as RNS temporal FRnet^26^ using the boundaries of the calculated stimulated brain regions. Lastly, we asked if these values differed in patients with a super responder (>90% seizure reduction) clinical outcome^26^.

## Methods

### Patients

Consecutive recordings selected from 14 patients who underwent intracranial monitoring with depth electrodes between 2014 and 2018 at the University of California Los Angeles (UCLA) and from 19 patients at the Thomas Jefferson University (TJU) in 2016–2018 for the purpose of localization of the SOZ. Data collection was planned before the study was conceptualized. Among these 28 patients, 18 underwent resections and ablations and 10 were implanted with RNS. Inclusion criteria for this study included pre-surgical MRI for MRI-guided stereotactic electrode implantation, as well as a post-implant CT scan to localize the electrodes, and stereo EEG recordings during non-rapid eye movement (REM) sleep at a 2 kHz sampling rate. Patients were excluded if: 1) no resection/ablation or RNS placement was performed; 2) a post-resection/ablation MRI or a post-RNS implant CT was not obtained; 3) no adequate postoperative clinical follow up; 4) A failure to record at least ten minutes of artifact free iEEG during non-REM sleep; and 5) graph theoretical analysis indicated incomplete or poor spatial sampling^18^. All patients provided verbal and written consent prior to participating in this research, which was approved by the UCLA and TJU institutional review boards. Eligible patients were identified through queries of pre-existing clinical databases. The epileptologist defined SOZ was aggregated across all these seizures during the entire iEEG evaluation for each patient^17^.

### Neuroimaging

Using an in-house pipeline (https://github.com/pennmem/neurorad_pipeline), T1- pre-implant and post-resection MRIs were obtained for each patient. Post-implantation SEEG and RNS CT scans were then co-registered and normalized with the MRIs using Advanced Neuroimaging Tools (ANTs)^27^ with neuroradiologist supervision. The position of each electrode contact in the post-SEEG implant CT and post-RNS placement CT was localized to normalized MNI coordinates and the Desikan-Killiany atlas^28^. Identification of the named electrode contacts in the resection cavity was performed manually in itk-SNAP.

### EEG recordings and HFO detection

Clinical iEEG (0.1–600 Hz; 2000 samples per second) was recorded from 8 to 16 depth electrodes, each with 7–15 contacts, using a Nihon-Kohden 256-channel JE-120 long-term monitoring system (Nihon-Kohden America, Foothill Ranch, CA, USA), for each patient. A larger number of electrodes with more contacts were implanted at TJU. For the recordings performed at UCLA. the reference signal used for was a scalp electrode position at Fz. The reference signal used for the TJU recordings was an electrode in the white matter. HFOs and sharp-spikes were detected in the non-REM sleep iEEG using previously published methods (https://github.com/shenweiss)^29–33^ implemented in Matlab (Mathworks, Natick, MA, USA) from 10–60 minutes, per patient^18^. All other programming, including the virtual resection simulations, was done using Matlab. Following automatic detection of HFO and sharp-spikes, false detections of clear muscle and mechanical artifact were deleted by visual review in Micromed Brainquick (Venice, Italy).

### Fast ripple derived. predictors of post-resection/ablation seizure outcome

The SOZ, FR (fast ripple on oscillation > 350 Hz, and all fast ripple on spike)^17,18,34^ and RonS resection ratio (RR) were derived as the number of SOZ contacts, FR or RonS events on removed channels divided by total number of SOZ contacts or events on all channels. All graph theoretical measures were calculated using the Brain Connectivity Toolbox (https://sites.google.com/site/bctnet/)^35^. The adjacency matrix for the spatial FRnet (FR rate–distance radius resected difference RDRRD) was calculated by the average rate (/min) of the events recorded by two respective nodes multiplied by the Euclidian distance (mm) between these nodes. The adjacency matrix for the mutual information (MI) networks were calculated using FR event ‘spike trains’ defined by the onset times of each event and then calculating MI between nodes using the adaptive partition using inter-spike intervals MI estimator.^36^ Using these adjacency matrices, and their inverses, the temporal FRnet A and B were calculated^18^. Support vector machines (SVM) were trained on the actual measures: 1) FR RR; 2) spatial FRnet; 3) temporal FRnet-A,B. The SVM was trained after normalizing the data and using a Radial Basis Function kernel that is automatically scaled to reduce the effect of outliers on SVM training^17^.

### Virtual Resections and Outcome Prediction

The initial virtual resection volume was determined by defining all the graph nodes (i.e., contacts) with a FR rate > 1/minute as a candidate set and finding the node with the smallest local efficiency (LE) as the candidate node in the candidate set. If no contacts exhibited a FR rate > 1 minute, or no such nodes remained in the candidate set, all nodes were included in the candidate set and the candidate node was selected as the node with the highest FR rate. The candidate node served as the center of the sphere of the virtual resection(s). A resection sphere with a 1 cm radius was initially simulated, centered on this initial candidate node, and all nodes falling within this sphere were included in the virtual resected set after excluding contralateral contacts. For all the nodes in the virtual resected set, the SOZ RR, RonS RR, FR RR, spatial FRnet, and temporal FRnet-A,B were calculated. Additionally, we quantified the proportion of overlapping and non-overlapping nodes in the virtual resected set and the set of nodes in the actual resection. Then, in the second iteration of the simulation, the node with second lowest LE, or second highest FR rate, was included in the resected set. The radius between the initial node and this second resected node, with an additional 1 cm buffer, was used to calculate a second sphere and define the new resected set. Iteration of the simulation continued through all the candidate nodes in the candidate set with an incrementally increasing, but not decreasing, radius. For each iteration of the simulation, the SVM predicted whether the outcome was seizure free.

### Virtual RNS stimulation lead placement

The 10 patients with RNS placement were subdivided into those with bilateral and unilateral placement of the RNS stimulation leads. For patients with bilateral placement, we defined two sets of nodes, for each hemisphere, with the highest FR rate. For patients with ipsilateral placement of the two stimulation leads, we defined two sets as ipsilateral contacts with the highest FR rate. We found the corresponding two sets of four “stimulation contacts” that generated FR at the highest rates. We then calculated the corresponding SOZ stimulation ratio, FR stimulation rate, and RNS temporal FRnet^21^.

## Results

### Patient characteristics

The study cohort for patients that underwent resections consisted of 23 patients, 12 males and 11 females, and excluded 36 other patients^18^. The patients had diverse etiologies with 6 of the 23 with normal MRI findings^18^. The seizures onset zones (SOZ) were localized to the mesial temporal lobe and cingulate cortex in 14 patients, lateral temporal lobe in 7, frontal lobe in 9, and parietal lobe in 3. Five patients had multilobar SOZs. Ten patients received an anterior temporal lobectomy and 2 patients laser ablation. In 6 of the 23 patients, the operation was a surgical revision. The post-operative seizure outcome was seizure free in 10 and non-seizure free in 13. Mean time at last follow up was 31.45 ± 3.1 months (standard error of the mean). The patient characteristics of the RNS patients are described in a previous study^21^, and among these ten patients 7 were intermediate responders and 3 were super responders^26^. Further details regarding the patients included in this study can be found in our prior publications^18,21^.

### Spatial sampling limitations

Among the 23 patients that underwent resections three of the non-seizure free patients exhibited incomplete spatial sampling. We defined incomplete spatial sampling as a failure to achieve seizure freedom despite resection of the entire FR network. We also found that one seizure free and one non-seizure patient with poor spatial sampling. In these patients, FR networks could not be characterized, and no electrode contact generated FR on spikes at a rate of > 1/min^18^. Since these five patients could confound SVM training they were excluded from the study.

### Support vector machine training

SVM training was performed using the FR RR, and FRnet measures of the remaining 18 patients, as well as their corresponding status as seizure free or not. 100-fold cross validation of the trained SVM demonstrated a loss of 0.22 equivalent to an accuracy of 0.78. We then tested the trained SVM on FR RR, and FRnet measures derived from virtual resections in individual patients.

### Virtual resections

**Figure 1:**
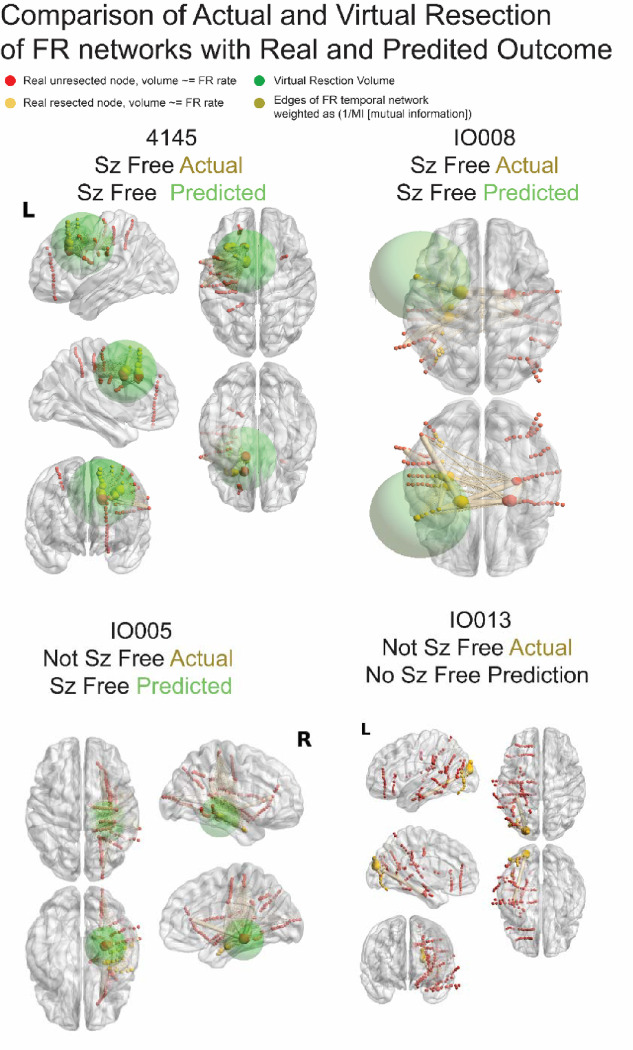
Illustration of fast ripple (FR) networks and real and simulated resections. In the four example patients, the sizes of red (unresected) and yellow (resected) nodes [i.e., stereo-EEG electrode contacts] are proportional to relative FR rate. The edges, connecting the nodes to one another, are weighted in size by the inverse of the mutual information (MI) of FR temporal correlations between the two nodes. In patients 4145 and IO008, who were actually rendered seizure free, the virtual resection overlaps strongly with the actually resected nodes. Also, the resection includes the nodes with the highest FR rates and connected by edges with the smallest MI. Patient IO005 was not actually rendered seizure free, the virtual resection that predicts seizure freedom is more posterior and includes nodes with high FR rate and low MI edges. Patient IO013 was also not rendered seizure free but the simulated virtual resection failed to achieve seizure freedom too.

**Figure 2:**
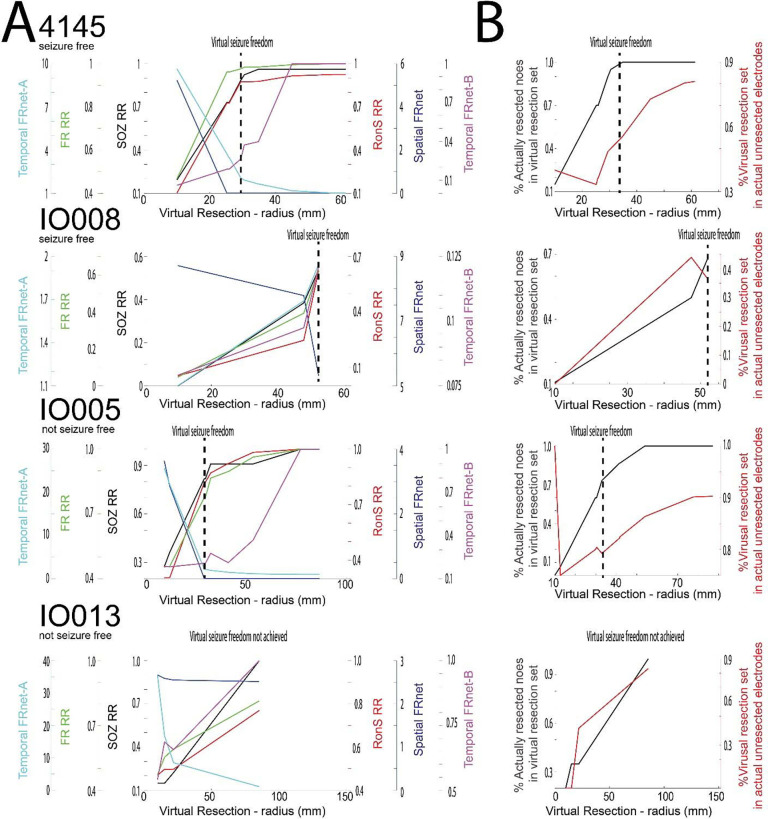
Changes in resection metrics in individual patients at different resection volumes. A) Comparison of the seizure onset zone resection ratio (SOZ, RR), ripple on spike RR (RonS RR, red), fast ripple RR (FR RR, green), spatial FRnet (blue), temporal FRnet-A (cyan), temporal FRnet-B (magenta) for two seizure free example patients (top), and two non-seizure free example patients (bottom). The hashed vertical line indicates the iteration at which virtual seizure freedom is first achieved. Note that, among the metrics, only lower spatial FRnet-A values (blue) are intrinsically associated with seizure outcome. Temporal FRnet-A values less than 1 are associated with non-seizure freedom, but small resections of desynchronous high rate FR nodes can result in FRnet-A values >> 1. Among the four patients, only in IO013 did a simulated resection fail to result in a seizure free outcome. B) Corresponding plot of the percentage of actually resected nodes in the resection set (black), and percentage of the virtual resection set in actual unresected nodes (red) for each of the four patients.

In the nine patients that were actually rendered seizure free, the simulated virtual resections, that achieved seizure freedom, almost always included all of the SOZ (mean SOZ RR = 0.87 ± 0.07) and most RonS (mean RonS RR = 0.76 ± 0.08) generating regions too (Table 1, Figure 1,2). These resections encompassed the actual resection contacts (86.7 ± 6.7% actual resection included in virtual resection) and included other unresected contacts too. Across the nine patients, 49.4 ± 8.0% of the contacts in the virtual resected were not actually resected. This implies that the virtual FR net resections required for virtual seizure freedom were larger than the actual resections of the SOZ that resulted in actual seizure freedom.

Among the nine patients not rendered seizure free by the actual surgery, five were rendered seizure free by virtual resections (Table 2, Figure 1,2). The virtual resections that rendered the patients virtually seizure free encompassed most of the SOZ (95.6 ± 4.5%) and RonS (87.1% ± 3.0%) generating regions. Overall, among the five patients who were not actually seizure free, but rendered seizure free by virtual resection, the virtual resections partially encompassed the contacts of the real resections (65.5 ± 19.3%). These virtual resections also included a substantial proportion of unresected contacts (69.6 ± 9.6%). Exceptions included patient IO015 who did not actually undergo a resection of the SOZ, and 4110 in which the virtual resection did not encompass most of the SOZ. Among the four patients who were not rendered seizure free by either the surgery or by virtual resections (Table 2, Figure 1,2) either the spatial FRnet value remained elevated and/or the temporal FRnet-B was relatively closer to zero.

### Power calculations for use of virtual resections in a randomized controlled clinical trial

Power calculations for a randomized controlled trial (RCT) to compare two approaches to epilepsy surgery: the standard resection of the seizure onset zone (SOZ), serving as the control arm, and an innovative approach where the standard resection is enhanced by FR net guided resections, designated as the active arm. The active arm employs the SVM model to predict whether a standard SOZ resection will result in the patient achieving seizure freedom. If the SVM model predicts that a standard resection is likely to fail, virtual resections that target specific areas identified by the FR net analysis amend the original surgical plan for the SOZ. The decision to proceed with the amended resection plan is contingent upon agreement from both the patient and the surgeon.

For this study, the sample size of 150 patients in each arm, amounting to a total of 300 participants, will provide 80% power to detect a difference of 0.15 in achieving seizure freedom rate between the two arms, under the assumption that the control arm exhibits a 60% seizure freedom and the active arm, benefitting from SVM-guided amendments, shows a significantly higher seizure freedom of 75%. This assumption is based on the preliminary results demonstrating an SVM classification accuracy of near 0.8 and guided amendments achieving seizure virtual seizure freedom in five of nine subjects. Two-sided Z-test with unpooled variance was used at a significance level of 0.05, to rigorously evaluate the efficacy of incorporating FR net analyses into surgical planning for epilepsy treatment. We anticipate a differential drop out rate due to: 1) an inability to fully resect the SOZ due to overlap with eloquent cortex; 2) refusal by patients or by physician for amended resections; 3) and patients that are lost to follow up with approximately 25% expected in the active arm compared 10% in the control arm. To account for this, 200 and 167 subjects in the active arm and control arm respectively may be enrolled to ensure a target sample size of 150/arm subjects post-drop out and attrition.

### RNS simulations

**Figure 3:**
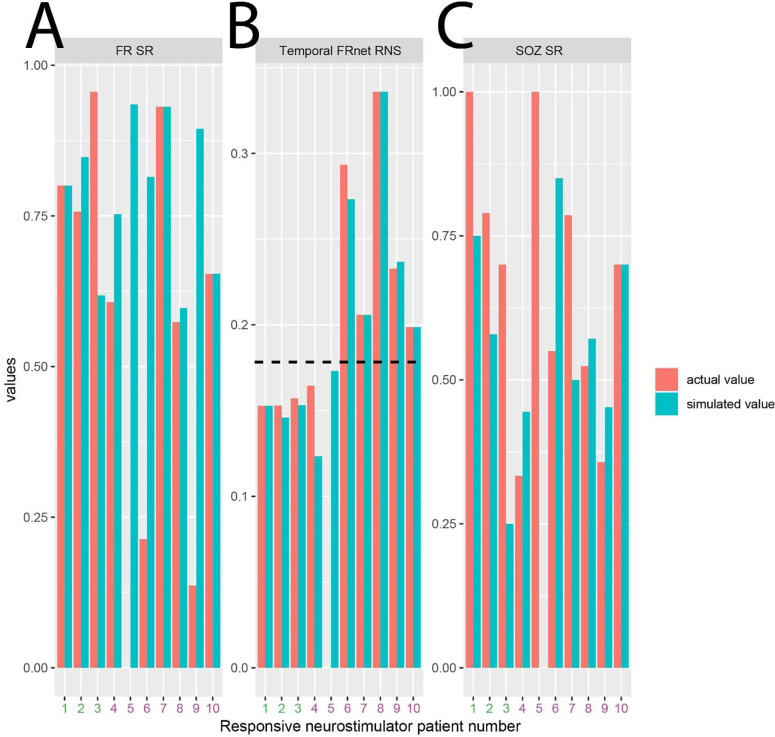
Simulated RNS lead placement and metrics that may predict RNS seizure outcome response. Fast ripple stimulation ratio (A, FR SR), temporal FRnet RNS (B, FR SGe), and SOZ stimulation ratio (C, SOZ SR) computed in patients 1–3 with a super responder outcome (green), and patients 4–10 with an intermediate responder outcome (magenta). In panel B note that patients 1–5 exhibited a simulated temporal FRnet RNS value less than patients 6–10 (horizontal hatched line). Placement of RNS leads in the simulated location thus may have resulted in five, rather than three, RNS super responder

Unlike the virtual resection experiments, we did not train a SVM to discriminate RNS super responders from RNS intermediate responders. However, in our prior study we had found that, among out 10 patients implanted with RNS, the RNS temporal FRnet value was smaller only in the three RNS super responder patients. We found that by virtually stimulating the nodes with the highest FR rates, the RNS temporal FRnet was substantially smaller in these three patients but also in two others (Figure 3). This suggests that targeting RNS stimulation to highly active FR generating tissue may increase the number of super responders.

Based on this preliminary simulation, we plan to enroll a total of 20 patients, divided equally into two groups: one active arm receiving RNS placement informed by our novel data analysis techniques, and one control arm subjected to standard RNS placement procedures. This sample size will achieve 80% power to detect a difference 0.5 between the group proportions of super responders. The proportion of super responders in the contr ol arm is assumed to be 0.3. The test statistic used is the two-sided Z-Test with unpooled variance at 0.05 significance level. A critical goal of this aim is to accurately estimate the effect size of our intervention, which is pivotal for the planning of future, more extensive research.

## Discussion

In summary, our stimulations show that geometrically simple (*i.e.,* spherical resections) that target FR networks, in most subjects, intrinsically overlap with regions denoted the SOZ and HFOs that demarcate the SOZ, such as RonS^37–39^. Most critically, in the patients rendered seizure free by the actual surgery, the curative virtual resections were substantially larger. The SOZ is defined using electroclinical correlation in the epilepsy monitoring unit^1^. Based on our modeling data, we propose that resection of the cortical territory delineated as the SOZ may be the minimal territory sufficient for achieving seizure freedom.

The virtual resections of the FR networks did successfully predict virtual seizure free in five of the nine patients who were not rendered seizure free by their actual resection. The simulated resections were larger than the actual resections and, in most cases, targeted the SOZ in addition to sites outside the SOZ generating FR at high rates and autonomously.

How can cortical territories outside the SOZ, that generate FR at high rates and autonomously, instigate seizures? We propose a seizure promoter zone in which synchronized neurons generate fast ripples in an autonomous fashion. These hyperexcitable events fail to elicit seizures because critical mass^40^ is not reached possibly due to inhibitory restraint^41–43^. We have shown previously that FR can propagate from the NSOZ to the SOZ and trigger epileptiform events there^44^. In the absence of a SOZ, FR originating from the seizure promoter region could trigger epileptiform spikes and seizures when they propagate to neighboring territories. Animal models of traumatic brain injury have also shown that, following the injury, fast ripples occur at higher rates and predict the occurrence of seizures weeks later^13^. Thus, following resection of the SOZ the seizure promoter zone could transform to the SOZ. Other important mechanisms that may be important in explaining the seizure promoter region include ictal wavefronts^45–47^, that are restrained in the promoter region, but upon propagation to a neighboring territory trigger seizure onset. A related mechanism may involve potassium efflux released by tonically active inhibitory neurons in the promoter region that results in a spreading depolarization^48^. When this spreading depolarization reaches the neighboring territory with less inhibitory restraint seizure onset is triggered.

Our power calculations indicate that our methods described herein could be utilized for a randomized controlled trial to improve resective epilepsy surgery outcomes. A larger retrospective study could be used to better train and test the LRM. A larger study would also identify the ideal inclusion and exclusion criteria. Due to sample size limitations, a clinical trial exploring alternative placement of RNS stimulation contacts is not yet feasible. Future studies could train and test a SVM that labels RNS super responders using SOZ stimulation ratio, FR stimulation ratio, and RNS temporal FRnet as factors. If this SVM’s accuracy is sufficient the SVM could be used to examine the virtual outcome resulting from virtual RNS stimulation electrode placements.

In summary our results utilizing virtual resections of FR networks indicate that the SOZ is the minimal cortical territory that must be resected to achieve seizure freedom. We propose that highly active and autonomous FR generating sites in FR networks demarcate both the SOZ and a seizure promoter region. In some patients the seizure promoter region may be found interweaved inside the SOZ, but in others it is outside the boundaries of the SOZ. Thus, we propose that using HFOs alone to plan a efficacious resection is maladaptive because a larger than needed territory will be targeted. Rather, FR networks can be used to predict the outcome of the resection of the SOZ and if needed identify the seizure promoter regions that also need be included to achieve seizure freedom.

## Figures and Tables

**Table 1:** Virtual resection metrics at which patients actually rendered seizure free by surgery were predicted to be rendered seizure free by the virtual resection. Abbreviations: SOZ: seizure onset zone; sim:simulation; RR:resection ration; FR; fast ripple: FRnet: FR network graph theoretical measure; percent_r: percent of the nodes in resection cavity included in virtual resection set; novel_r: percent of nodes in the resection set that were not in the resection cavity.

Patient	sim. Radius	SOZ RR	RonS RR	FRRR	spatial FRnet	temporal FRnet-A	temporal FRnet-B	percent_r	novel_r
1) 466	45.907	1.000	0.593	0.459	1.869	0.874	0.539	1.000	0.548
2) 477	18.565	1.000	0.880	0.845	3.561	3.905	0.406	1.000	0.000
3) IO018	44.094	0.563	0.750	0.661	0.666	2.376	0.545	0.406	0.395
4) 4145	29.417	0.885	0.874	0.976	0.000	2.062	0.264	0.900	0.486
5) 4124	28.905	1.000	0.959	0.652	1.841	1.861	1.000	0.909	0.730
6) 4166	84.966	1.000	1.000	1.000	0.000	1.000	1.000	1.000	0.639
7) IO008	51.888	0.538	0.623	0.627	5.312	1.946	0.121	0.679	0.367
8) IO001	45.379	1.000	0.882	0.800	0.978	1.622	1.000	0.909	0.836
9) 453	72.011	1.000	0.263	0.589	0.000	1.279	1.110	1.000	0.452

**Table 2:** Virtual resection metrics at which patients not actually rendered seizure free by surgery were predicted to be rendered seizure free by the virtual resection. Some patients did not achieve seizure freedom in the simulation (red). Abbreviations: SOZ: seizure onset zone; sim:simulation; RR:resection ration; FR; fast ripple: FRnet: FR network graph theoretical measure; percent_r: percent of the nodes in resection cavity included in virtual resection set; novel_r: percent of nodes in the resection set that were not in the resection cavity.

Patient	sim. Radius	SOZ RR	RonS RR	FRRR	spatial FRnet	temporal FRnet-A	temporal FRnet-B	percent_r	novel_r
10) IO005	29.848	0.818	0.821	0.776	0.000	2.222	0.207	0.600	0.800
11) IO012	18.417	1.000	0.879	0.737	3.083	4.898	0.084	0.818	0.471
12) 469	53.053	1.000	0.824	0.947	0.000	1.249	0.353	0.857	0.478
13) 4110	35.077	0.318	0.191	0.833	0.000	1.282	0.088	0.162	0.905
14) 462	75.660	1.000	0.906	0.429	4.360	0.894	0.262	1.000	0.600
15) IO023	74.120	1.000	0.935	0.942	0.000	1.206	0.096	0.000	1.000
16) IO013	85.596	1.000	0.769	0.814	2.517	1.076	1.000	1.000	0.832
17) IO015	50.239	0.625	0.992	0.915	3.261	1.629	0.099	0.000	1.000
18) IO018	64.902	1.000	0.998	0.932	2.602	1.001	1.000	1.000	0.589

## Data Availability

The datasets generated during and/or analysed during the current study are available from the corresponding author on reasonable request.
